# Esophagitis Dissecans Superficialis in a Patient With Prior Subtotal Gastrectomy and Roux-en-Y Reconstruction: A Case Report

**DOI:** 10.7759/cureus.115243

**Published:** 2026-08-26

**Authors:** Ruth T Samson, Julia Schroer, Duane E Deivert, Alexandra Falvo

**Affiliations:** 1 Breast Surgery, Montefiore Medical Center, Bronx, USA; 2 General Surgery, Houston Methodist, Houston, USA; 3 Gastroenterology, Geisinger Health System, Wilkes-Barre, USA; 4 Bariatric and Foregut Surgery, Geisinger Health System, Scranton, USA

**Keywords:** case report, dysphagia, esophageal mucosal injury, esophagitis dissecans superficialis, sloughing esophagitis, upper gastrointestinal endoscopy

## Abstract

Esophagitis dissecans superficialis (EDS), also known as sloughing esophagitis, is a rare, benign disorder characterized by superficial sloughing of the esophageal mucosa. Its pathogenesis remains incompletely understood, with several underlying conditions and medication exposures described as potential predisposing factors. We present the case of a 41-year-old woman with an extensive surgical history, including subtotal gastrectomy with Roux-en-Y reconstruction, who presented with chronic upper abdominal pain and bloating. Esophagogastroduodenoscopy demonstrated superficial sloughing of the esophageal mucosa consistent with EDS. Subsequent histopathologic examination confirmed the diagnosis and showed no evidence of fungal infection. Because esophageal candidiasis was initially suspected based on the endoscopic appearance, the patient was empirically treated with fluconazole and continued proton pump inhibitor therapy. Repeat endoscopy six months later demonstrated complete resolution of the esophageal abnormalities. This case highlights EDS in a patient with extensively altered foregut anatomy and illustrates its potential to mimic infectious esophagitis. Although a causal relationship between prior gastric reconstruction and EDS cannot be established, recognizing this entity and correlating endoscopic and histopathologic findings are important for accurate diagnosis.

## Introduction

Esophagitis dissecans superficialis (EDS), also called sloughing esophagitis, is an uncommon, benign disorder characterized by superficial desquamation and shedding of the esophageal mucosa [1-4]. Its true prevalence is uncertain, in part because patients may be asymptomatic and the condition may be overlooked or mistaken for other esophageal disorders. In a retrospective review of 21,497 upper endoscopies, Fiani et al. identified EDS in seven patients, corresponding to a detection rate of 0.03% [4].

The etiology of EDS remains incompletely understood. Reported associations include chronic debilitation, malnutrition, polypharmacy, medications capable of causing esophageal mucosal injury, autoimmune bullous dermatoses, and disorders associated with mechanical or chemical injury to the esophagus [1,3,5-8]. Despite its striking endoscopic appearance, EDS generally follows a benign course.

We present a case of EDS in a 41-year-old woman with a complex surgical history involving multiple gastric operations culminating in subtotal gastrectomy with Roux-en-Y reconstruction. This case highlights the diagnostic challenge of distinguishing EDS from infectious esophagitis and discusses the potential relevance of extensively altered foregut anatomy in a patient with confirmed EDS.

## Case presentation

A 41-year-old woman with a history of gastroparesis, gastroesophageal reflux disease, chronic nonhealing peptic ulcer disease, gastric outlet obstruction, ulcerative colitis, recurrent gastroenteritis, anxiety, and depression presented with chronic upper abdominal pain and bloating.

Her surgical history was extensive and included approximately 11 abdominal operations. More than 10 years earlier, she underwent laparoscopic partial gastrectomy with Billroth I reconstruction; the indication for this index operation could not be confirmed from the available records. Subsequent revisions ultimately resulted in subtotal gastrectomy with Roux-en-Y reconstruction. Her postoperative course was complicated by a duodenal stump leak and intra-abdominal abscess formation requiring multiple laparotomies for drainage and placement of a jejunal feeding tube. She remained on enteral feeding until oral intake improved sufficiently for tube removal. Despite treatment with a proton pump inhibitor and prokinetic therapy, she experienced ongoing nonspecific abdominal symptoms. A previous esophagogastroduodenoscopy (EGD) had demonstrated normal esophageal mucosa and an intact gastric pouch. She was subsequently treated with omeprazole 40 mg twice daily and simethicone 125 mg four times daily.

The patient was a former smoker with an 11-pack-year history but denied regular alcohol consumption and use of carbonated beverages, coffee, or tea. Her medications included omeprazole, simethicone, promethazine, ondansetron, prochlorperazine, bisacodyl, acetaminophen, and hydroxyzine. She denied nonsteroidal anti-inflammatory drug or bisphosphonate use.

On evaluation, the patient appeared comfortable and reported epigastric discomfort and abdominal fullness. She denied weight loss, dysphagia, odynophagia, vomiting, changes in bowel habits, melena, or hematochezia. Her vital signs were within normal limits. Abdominal examination demonstrated mild distention and minimal epigastric tenderness without peritoneal signs. Multiple well-healed surgical scars were present.

**Figure 1:**
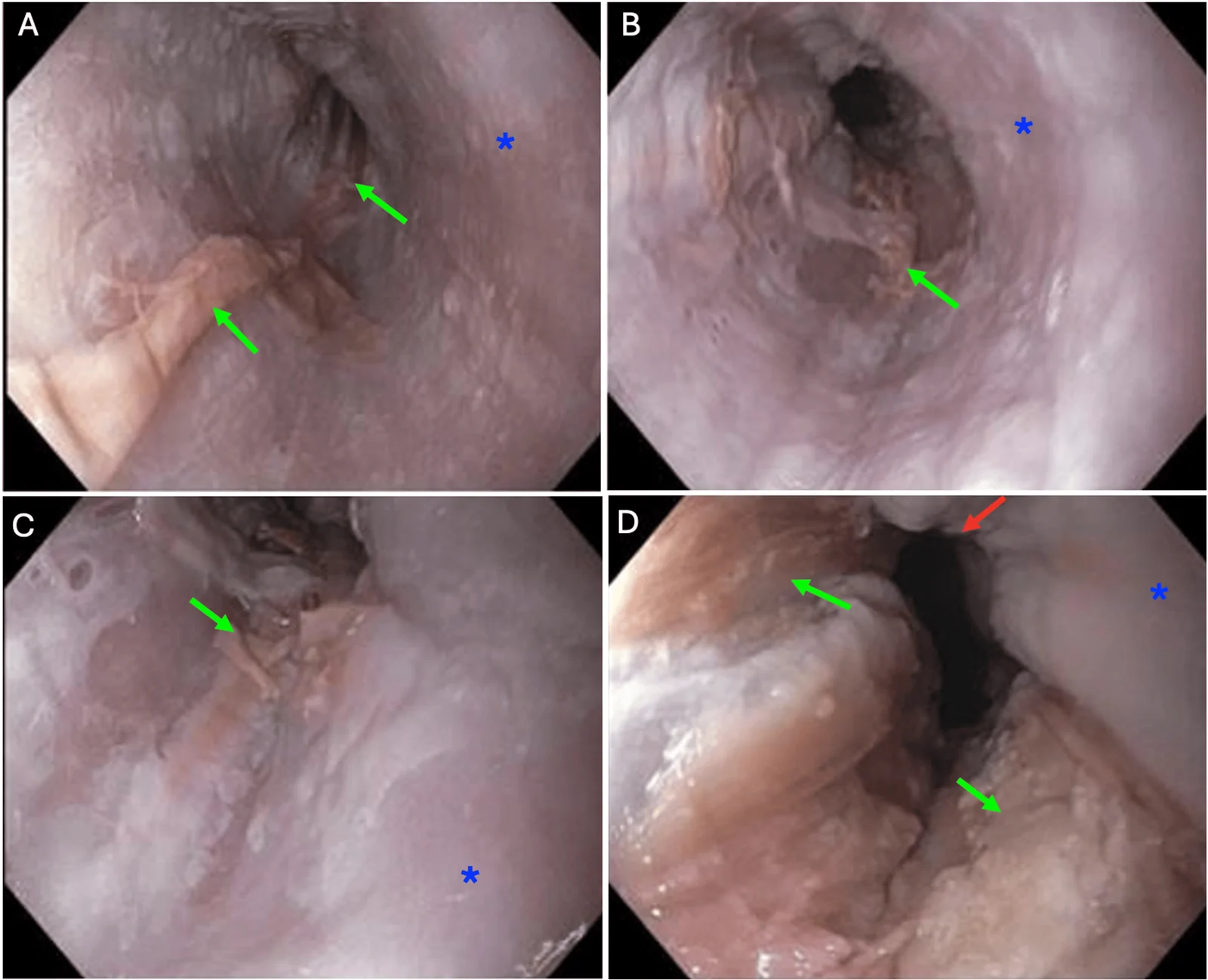
Endoscopic findings of esophagitis dissecans superficialis. (A-C) Endoscopic images demonstrating mucosal sloughing and desquamation extending from the mid-to-distal esophagus. Green arrows indicate areas of sloughed/desquamated mucosa, while blue asterisks indicate areas of spared normal-appearing esophageal mucosa. (D) Extensive mucosal desquamation of the distal esophagus near the gastroesophageal junction, indicated by the red arrow.

Given her persistent symptoms, repeat EGD was performed (Figure 1). Examination demonstrated linear strips and sheets of pale, gray-white superficial mucosa sloughing from the mid-to-distal esophagus, with grossly normal-appearing underlying mucosa and no significant bleeding or ulceration. The gastric pouch and gastrojejunal anastomosis appeared healthy, without ulceration or stenosis. These esophageal abnormalities were new compared with prior endoscopic examinations.

Biopsies were obtained from areas of mucosal sloughing. Histopathologic examination demonstrated two-toned squamous epithelium with superficial parakeratosis, scattered pyknotic and ghost keratinocyte nuclei, and an intraepithelial cleavage plane separating the necrotic superficial layer from viable underlying epithelium. Adherent Gram-positive cocci were present along the sloughed fragment without associated acute inflammation. Periodic acid-Schiff and Grocott methenamine silver stains showed no fungal organisms. Correlative tissue culture grew mixed oropharyngeal/commensal bacterial flora without fungal growth. These findings were diagnostic of EDS.

Given initial concern for esophageal candidiasis based on the endoscopic appearance, the patient was empirically treated with oral fluconazole 400 mg daily and continued proton pump inhibitor therapy. Repeat EGD six months later demonstrated healthy-appearing esophageal mucosa with complete resolution of the previously observed mucosal sloughing.

## Discussion

EDS is an uncommon esophageal disorder characterized endoscopically by sloughing of the superficial mucosa, which may appear as white strips, patches, or sheets of detached tissue [1-4]. Histologic findings include superficial epithelial necrosis and splitting, parakeratosis, and variable inflammation, although histopathologic abnormalities may occasionally be nonspecific [1,3,5]. Ponsot et al. described mucosal blistering with reduced numbers of desmosomes and decreased immunoreactive E-cadherin expression, suggesting that impaired intercellular adhesion may contribute to epithelial sloughing [6]. Special stains such as periodic acid-Schiff (PAS) or Grocott methenamine silver (GMS) may help exclude fungal infection when clinically suspected rather than establishing EDS itself. Recognition of the characteristic endoscopic appearance, histopathologic evaluation, and exclusion of competing causes of esophageal mucosal injury are therefore important components of the diagnostic evaluation.

Clinical presentation is variable. Patients may be asymptomatic or may present with dysphagia, odynophagia, nausea, vomiting, abdominal or epigastric pain, gastrointestinal bleeding, or weight loss [1,3,4]. Because of both its rarity and variable presentation, EDS may be under-recognized during upper endoscopy.

Several predisposing conditions have been reported. In their clinicopathologic series, Purdy et al. found EDS to be associated with chronic debilitation and the use of multiple medications, particularly medications capable of injuring the esophageal mucosa [1]. Carmack et al. similarly demonstrated substantial clinical heterogeneity among patients with EDS [3]. Autoimmune bullous disorders, including pemphigus vulgaris and bullous pemphigoid, have also been associated with EDS [7]. Other reported associations include celiac disease, esophageal strictures, and esophageal motility disorders [8-10].

Medication-related mucosal injury is another important consideration. EDS has been described in association with bisphosphonates, oral iron supplementation, clindamycin, and methotrexate toxicity [11-14]. The patient in the present case denied nonsteroidal anti-inflammatory drug and bisphosphonate use and was not documented to be receiving these previously reported offending medications. However, she had several other potential predisposing features, including chronic gastrointestinal disease, polypharmacy, and an extensive history of foregut surgery.

The patient in this case had markedly altered gastrointestinal anatomy, which is an additional clinical consideration. She underwent multiple gastric operations culminating in subtotal gastrectomy with Roux-en-Y reconstruction and experienced substantial postoperative morbidity requiring additional laparotomies and temporary enteral nutritional support. Gastric resection and reconstruction can alter gastrointestinal motility, luminal transit, and exposure of the esophagus to gastric and intestinal contents. Postgastrectomy esophagitis has been associated with exposure to conjugated bile acids and pancreatic enzymes such as trypsin, demonstrating that esophageal mucosal injury after gastric surgery is not necessarily mediated by acid alone [15]. Roux-en-Y reconstruction may reduce pancreatobiliary reflux by diverting these contents distally, although the degree of diversion depends on the reconstruction [15,16].

The relevance of this altered anatomy to EDS remains uncertain. Esophageal mucosal integrity reflects a balance between luminal injury and epithelial defense and repair. Extensive foregut reconstruction may substantially alter the physiologic environment of the esophagus, providing a plausible clinical context in which superficial epithelial injury could occur. However, a direct causal relationship between subtotal gastrectomy, Roux-en-Y reconstruction, and EDS has not been established, and this case cannot determine whether the patient's surgically altered anatomy contributed to the development of EDS.

An important diagnostic consideration in this case was esophageal candidiasis, as the white, sloughing mucosal appearance of EDS may mimic Candida esophagitis and other inflammatory, infectious, or neoplastic processes [3,17]. De and Williams similarly described EDS in a patient initially treated for Candida esophagitis, highlighting the potential diagnostic overlap between these entities [17]. In the present case, the endoscopic appearance prompted empiric treatment with fluconazole; however, histopathologic examination demonstrated characteristic features of EDS, including superficial parakeratosis, necrotic keratinocytes, and intraepithelial cleavage. PAS and GMS stains showed no fungal organisms, and tissue culture demonstrated no fungal growth, supporting EDS rather than Candida esophagitis. Although the esophageal abnormalities had resolved on repeat endoscopy six months later, resolution cannot be attributed specifically to antifungal therapy given the absence of demonstrated fungal infection, concurrent proton pump inhibitor therapy, and the frequently benign and self-limited course of EDS.

There are currently no standardized treatment guidelines for EDS. Management generally focuses on eliminating potential mucosal insults and treating associated conditions. Proton pump inhibitors are frequently used, particularly when acid-mediated mucosal injury is suspected, although their role may primarily involve protection from additional injury rather than treatment of a specific underlying mechanism [4,18]. Corticosteroid therapy has been reported in severe cases or in association with immune-mediated disease [18]. Most reported cases demonstrate a favorable clinical course, with eventual improvement or resolution of endoscopic abnormalities [1,3,4,18].

## Conclusions

EDS is a rare and under-recognized benign disorder characterized by superficial sloughing of the esophageal mucosa. In this patient, characteristic endoscopic findings together with diagnostic histopathology established EDS and helped distinguish it from Candida esophagitis. Although this case cannot establish a causal relationship between altered surgical anatomy and EDS, it demonstrates EDS in the setting of extensive foregut reconstruction following subtotal gastrectomy with Roux-en-Y reconstruction.

Careful correlation of endoscopic, histopathologic, and clinical findings is important when evaluating suspected EDS and excluding alternative causes of esophageal mucosal injury. Although the mucosal abnormalities resolved after empiric fluconazole therapy, fungal infection was not demonstrated, and the resolution cannot be attributed specifically to antifungal treatment given the frequently self-limited course of EDS. Further investigation is needed to determine whether altered postoperative gastrointestinal anatomy and physiology contribute to susceptibility to EDS.
